# Supplementary material for: Quantitative principles of cis-translational control by general mRNA sequence features in eukaryotes
Source: Genome Biol. 2019 Aug 9;20:162. doi: 10.1186/s13059-019-1761-9 (PMC6689182; doi:10.1186/s13059-019-1761-9)
Supplement: Supplementary file 11 — Review history. (DOCX 36 kb) [file 13059_2019_1761_MOESM11_ESM.docx]

Review History

**First round of review**

**Reviewer 1**

**Are you able to assess all statistics in the manuscript, including the appropriateness of statistical tests used?**

Yes, and I have assessed the statistics in my report.

**Comments to author:**

As a central process in gene expression, translational control is extremely important to biological organisms. A large body of work has previously identified sequence and structural features of 5' UTRs and CDS coding regions that correlate with translation efficiency in eukaryotes, owing to the demands of directional scanning in translation initiation and codon usage bias in translation elongation. Li et al. present a manuscript comparing correlative analyses of these features with ribosome occupancy data from five organisms. This follows up on their previous work evaluating correlations between features and ribosome occupancy in S. cerevisiae. Interestingly, they find that predicted RNA structure and sequence composition within 5' UTRs both correlate well with estimated ribosome occupancy in all five species, and that these features co-correlate (such that their effects are not completely separable). They also find species differences in 5' UTR sequence composition that may contribute to differences in the magnitude of cis-acting sequence control on mRNA translation among species. Finally, they compare the While the subject is timely and some of the results are interesting, I have significant concerns about differences in datasets used here for the purposes of comparing across species. Because the datasets were not analyzed in a consistent way and the features used vary among species, comparisons among datasets may be biased. There are also concerns about the broad scope of the discussion and lack of presentation of alternative interpretations. Individual issues follow:

Approach and results issues:

1) Datasets from different species are not processed equally, as the data were taken directly from paper supplemental tables. Alternative splicing in metazoans may be drastically changing the G/C content if not properly accommodated. Most published ribosome profiling studies fudge this in metazoans due to the short length of ribosome footprints, which result in many "multi map" reads. This decreases the fidelity of ribosome profiling estimates in these species. The authors should use a consistent data processing pipeline that consistently establishes the expressed mRNA isoforms in each of the metazoan datasets and deals appropriately with multi-mapping of short ribo-seq reads.

2) The genome annotations in metazoans are much more complicated than those in simple eukaryotes like S. cerevisiae and S. pombe. The authors don't describe in the main text which annotations were used or how 5' UTR sequences were obtained. Indeed, many genes have alternative 5' UTRs and it would be vital to use the 5' UTR isoforms expressed in each experiment for correlative analysis with the corresponding ribosome occupancy. This also underscores the importance of consistent data processing.

3) Datasets from different species have differing amounts of polyA bias. The Weinberg S. cerevisiae dataset has essentially no polyA bias by using Ribozero to estimate mRNA levels. This led to very different ribosome occupancy estimates. Indeed, it decreases the translation efficiency (ribosome occupancy) variance among genes in S. cerevisiae. What about the other datasets? Do they have more apparent variance among translation efficiency? This might help explain why less of the variance is captured by the regression models for the other species and datasets, compared to the Weinberg data.

4) Ribosome profiling does not provide translation efficiency estimates. It provides ribosome occupancy estimates only. The dataset from S. cerevisiae tried to account for this by taking into consideration codon usage. It has Initiation Rates estimates, while the other datasets have ribosome occupancy only. Even so, one can't exactly equate the two things. The authors should note this caveat somewhere in the introduction or results.

5) As above, the yeast dataset has codon usage baked into the "initiation rates" (Weinberg et al), while the other species datasets do not. Does this account for the fact that the models recover more of the variance in yeast data? Since the impact of codon usage is already included into the initiation rate estimates, is it fair to use codon usage as a predictor for these initiation rates in S. cerevisiae?

6) The authors use "translation rates (e.g. page 20, Line 11) when they mean translation efficiency. "Translation rates" are the number of proteins translated per unit time. Translation rates (estimated by ribosome occupancy) correlate quite well with protein abundance (in contrast to author's claim on pg 20 line 12). I realized later on that they defined "translation rate" in their earlier NAR publication this way, but didn't see it defined in this manuscript.

7) The AUG Proximal Element regions (APEs) vary too much between species to allow meaningful across-species comparisons. Typically, this region is thought to comprise the "Kozak" sequence (-5 to +3), but the authors have allowed it to occupy much more sequence in S. cerevisiae and nearly the entire UTR region in A. thaliana. How do the results change if you consider only the Kozak region, or same sized regions as APEs?

Discussion section issues:

8) The higher G/C content in mammalian 5' UTRs might be explainable simply as a difference in growth temperature, as the other species must accommodate shifts in growth temperature that alter the stability of 5' UTR structures (especially at low temperature), while mammals maintain a constant, relatively elevated temperature. This could be discussed, but may have been covered by previous work (e.g. by Michael Lynch).

9) It is true that RNA abundance correlates fairly well overall with protein abundance. However - codon usage influences mRNA abundance by affecting RNA decay rates (Coller, Green, and others). It is thus wrong to claim that translation rates correlate poorly with protein abundance and that transcriptional control is the primary influence. Furthermore, recent work has linked RNA decay with upregulated transcription in mice and zebrafish (Nonsense Induced Transcription Compensation, Wilkinson et al. N&V, 2019), echoing older work from yeast (Choder lab). Thus transcription rates are influenced by translation rates. The connections between these processes complicate comparisons of the influence of transcription and translation on protein production. The authors should present a more balanced view on this topic in the discussion, describing such caveats.

10) Also, statements about the lack of importance in translational control made in the discussion are based entirely on correlative analyses of steady-state measurements in single-cell organisms and tissue culture cells. Translational control is important for organismal development, meiosis, local protein production (e.g. at the ends of neuronal axons), immune responses, general stress responses, etc. The authors should note their results are based on steady-state cell growth conditions, and thus miss many aspects of translational control somewhere in their discussion, which currently comes off as biased against any important role for translational control in biological organisms.

12) The evolutionary arguments in the discussion are also highly biased against translation and don't consider alternatives. For example, "...the length of a protein is unlikely to be selected only to affect translation. Protein length is presumably set largely by protein function" (pg 20 line 13). The opposite point could be argued. Housekeeping proteins are small, often splitting single functions into multi-domain complexes. One benefit of keeping such proteins small is that they are more efficiently translated. Similarly, "Translation rates are determined by multiple selective pressures, In contrast, the selective pressure on protein abundance dominantly affects transcription"(pg 21 line 1). One could argue that mutations that decrease translation rates have fitness effects and many features of 5' UTRs are highly conserved.

12) Underscoring the counterarguments against the evolutionary statements in the discussion, She and Jarosz recently mapped hundreds of causal nucleotides responsible for natural phenotypic variation in yeast, and found that causal variants in 5' UTRs, Synonymous changes, and 3' UTRs were more numerous than variants in intergenic regions (Cell 2018). Mutations that introduce uAUGs are linked to numerous human genetic disorders. Thus there is ample evidence for translation efficiency variation affecting phenotype and being subjected to negative selection.

13) The authors results suggest that single nucleotide variations in 5' UTRs could drastically shift translation efficiency (pg 17, line 3) by altering RNA structure. This also supports the above arguments that changes in 5' UTR sequences likely alter phenotypes and are likely subject to natural selection.

14) Pg 21, line 16 "mutations" should be "sequences", as these were not changes from a wild type, but rather random sequences cloned in the 5' region.

15) Pg 22, line 11 "...measured endogenous translation rates". The authors should note that they aren't using translation rates, they are using yeast initiation rates estimated from ribosome profiling after accounting for codon effects.

16) The manuscript should be reorganized to reduce the number of main figures (17), and reduce the length.

**Reviewer 2**

**Are you able to assess all statistics in the manuscript, including the appropriateness of statistical tests used?**

There are no statistics in the manuscript.

**Comments to author:**

Review of Biggen et al. "Quantitative Principles of cis-translational control by general mRNA sequence features in eukaryotes"

mRNAs encode many cis-regulatory elements that have the potential to control the rate of protein synthesis. Such elements include secondary structures, uAUGs & uORFs, start codon context, CDS length, and codon usage. While the role of individual elements has been demonstrated on particular mRNAs, computational models that integrate each element to account for differences in translation efficiency are lacking or have poor predictive power. Here Li et al report an integrated model that accounts for about 40-80% of the translation rate (TR) variance in five species: S. cerevisiae, S. pombe, A. thaliana, M. musculus, and H. sapiens. They demonstrate that the stability of the most stable secondary structure in a 5' region of a gene has predictive power for TR across all species except S. pombe. The location of the most stable secondary structure varies among species, either near the 5' cap (mammals) or the start codon (the others). They further show that across all five organisms, changes in tri-nucleotide frequencies correlate with TR. By integrating models that account for the 5' region features, codon frequency, and CDS length they yielded a model that explains 37-81% of the variance in TR. Lastly, they demonstrate that 5' region features, codon frequency, and CDS length are collinear suggesting that these features are coordinated to control ribosome density on mRNAs.

Despite its potentially interesting findings and its clarity, I cannot recommend publication of this manuscript in Genome Biology at this point. One hesitation is that they have previously published a model (Li et al. 2017. NAR.) that accounted for 80% of the TR variance in S. cerevisiae. It is unclear how the model/approach they present here is different from their previous work since the reported predictive power is identical to the what they have previously reported for S. cerevisiae. My interpretation is that it is quite similar or even identical. For instance, the S.cerevisiae data included in Figure 7 appears identical to data they have previously published (Li et al. 2017. NAR, Figure 8A,C). If so, this must be qualified and cited. Further, much of Figures 2-12 could be condensed and summarized as a few key figures in the main text, with much of it instead included in the supplement. I think the authors make interesting points that are obfuscated by the amount of analyses that surround them (particularly Figures 3 & 9). Summary diagrams that outline species specific findings would also be helpful.

It is interesting that a majority of the variance in TR in the examined single-celled organisms can be explained by the modeled general features, while only a minority is explained in multicellular organisms. Given their discussion points on the role of effective population size, the manuscript would be improved if the authors demonstrated that this observation held true for another non-fungal single-celled eukaryote. As is, the single-celled organisms they have profiled represent a different kingdom of life than the multi-cellular organisms they selected, which may limit the strength of their conclusions. One intriguing candidate would be Chlamydomonas reinhardtii, for which ribosome profiling data is available (PMID: 26286745) and would offer a useful comparison to Arabidopsis.

Further comments that should be addressed

1. The authors should describe how the example datasets were chosen, and whether the experiments were conducted using similar protocols (e.g. identical translation inhibitors, etc.). These data are the foundation of the manuscript and it is important to disclose potential experimental biases among the datasets.

2. In Figure 2 it is unclear why the authors chose to group S. pombe with S. cerevisiae and Arabidopsis given that High TR genes appear to have more stable secondary structures from +1 to +30, which contrasts with the other species. They should comment on this, especially since on page 6 lines 12-14 they state "Given this and a lack of evidence that RNA structure affects the elongating ribosome, we have limited our models to the 5'UTR and the 5 most part of CDS, where folding energy values and translation rates correlate positively." For S. pombe, it appears that more stable secondary structures just downstream of the start codon are correlated with higher translation efficiencies.

3. Figure 3 should be distilled, with most of the data included in the supplement. While the authors refer back to this figure at different points in the results, it is initially overwhelming and quite difficult to interpret. I would suggest a single R2 feature vs TR plot for each species that displays "whole", "RNA fold", and a subset of the previously used models as a comparison.

4. Figure 7a. The y-axis label should be "probability" rather than "bits". In these plots it is difficult to visualize the differences between the high and low groups among species. It may help to generate PWMs of high TR groups and use the low TR groups as the background, and vice versa. This would highlight enriched features in each group relative to the other.

Also, the authors don't seem to account for or acknowledge the potential role of the kozak sequence within the APEs (Results, Sequence motifs in 5' UTRs). I suspect this will account for at least some of the variance in TR. For instance, in high TR genes from S. cerevisiae adenosine is enriched at the -3 position (relative to AUG), which matches the canonical kozak sequence in this species. The same is true for S. pombe, but it appears less so for the mammalian species. While they allude to the kozak sequence (I think) later on page 17 line 23, it would be helpful to discuss it during the section with the enriched sequence elements.

**Authors Response**

**Point-by-point responses to the reviewers’ comments:**

*We are extremely grateful to both reviewers for taking the time to read our manuscript, for their stimulating and thoughtful comments, and for recognizing that our work is of interest. In response we have included an additional analysis that shows that any variation in translation rates between mRNA isoforms is too small to impact our conclusions: We show that we obtain substantially similar results when we limit our analysis to those genes that have only a single mRNA isoform in Arabidopsis and mouse. In addition, we have modified the text to clarify the methods used and to better explain our conclusions. Below we address each of the reviewer’s comments in turn. Changes in the text of the manuscript are shown in track changes in a version of the paper in the supplementary materials.*

Reviewer #1:

As a central process in gene expression, translational control is extremely important to biological organisms. A large body of work has previously identified sequence and structural features of 5' UTRs and CDS coding regions that correlate with translation efficiency in eukaryotes, owing to the demands of directional scanning in translation initiation and codon usage bias in translation elongation. Li et al. present a manuscript comparing correlative analyses of these features with ribosome occupancy data from five organisms. This follows up on their previous work evaluating correlations between features and ribosome occupancy in S. cerevisiae. Interestingly, they find that predicted RNA structure and sequence composition within 5' UTRs both correlate well with estimated ribosome occupancy in all five species, and that these features co-correlate (such that their effects are not completely separable). They also find species differences in 5' UTR sequence composition that may contribute to differences in the magnitude of cis-acting sequence control on mRNA translation among species. Finally, they compare the While the subject is timely and some of the results are interesting, I have significant concerns about differences in datasets used here for the purposes of comparing across species. Because the datasets were not analyzed in a consistent way and the features used vary among species, comparisons among datasets may be biased. There are also concerns about the broad scope of the discussion and lack of presentation of alternative interpretations. Individual issues follow:

Approach and results issues:

1) Datasets from different species are not processed equally, as the data were taken directly from paper supplemental tables. Alternative splicing in metazoans may be drastically changing the G/C content if not properly accommodated. Most published ribosome profiling studies fudge this in metazoans due to the short length of ribosome footprints, which result in many "multi map" reads. This decreases the fidelity of ribosome profiling estimates in these species. The authors should use a consistent data processing pipeline that consistently establishes the expressed mRNA isoforms in each of the metazoan datasets and deals appropriately with multi-mapping of short ribo-seq reads.

*RESPONSE*

*We thank the reviewer for this comment. We would first like to explain why we designed the analysis pipeline presented our paper. Our analysis pipeline is the same for all species and datasets. Its great strength is its robustness to the large differences in GC contents of eukaryotic genomes, changes in the wet lab protocols used to generate the ribosome profiling and mRNA abundance data, and any differences in how sequence reads were mapped to genomes etc. by the authors of the original papers. The major conclusions of our manuscript (i.e. those in the Abstract) are based on observations that are common across all species and datasets. Results for datasets from the same species are particularly consistent, despite the fact that the datasets in each of these species were generated by two separate laboratories and represent different tissues or conditions and protocol variants. Further, one of the datasets from S, cerevisae, S. pombe, and M. musculus was produced by a single graduate student using an (almost) consistent pipeline (work from Weinberg et al. and Subtelny et al.). Hence, our findings suggest that any variation due to GC content, wet lab protocols, or sequence read mapping are very unlikely to affect the major claims of our manuscript.*

*Based on our understanding, the reviewer made two points: (1) we should use a consistent pipeline to map sequence reads and process the data; (2) we should control for variation in mRNA isoforms. Below we discuss each of them.*

*For point (1), we do not expect that re-mapping sequence reads to the genome by a common pipeline would improve the analysis. In addition to the fact that our conclusions are robust to the variation in read mapping protocols (see above), a re-mapping using the same set of parameters may actually have a negative impact. The sequence reads were generated by different protocols and machines and also have different GC contents, sequencing errors, etc. The degree of RNA digestion (footprint length) varies too, quite a bit in the case of the Arabidopsis data we used from two groups. Hence, using a common set of read mapping parameters could impose different degrees of mapping errors, which would be unnecessary and undesirable for our analysis. Instead, we decided to take data from quality publications that have been analyzed and shown by their authors (given the particular mapping parameters each investigator has chosen) to pass various quality control tests, including having a strong bias for the expected positioning of ribosomes at the first nucleotide of each codon. These authors are far more aware of the details of their experimental protocols than we, and in our view they are best placed to decide the mapping parameters. For the above reasons, we have decided not to re-do the mapping using our own criteria as this would be unlikely to be optimized for each dataset. Instead, we had previously added filtering steps to some of the datasets to bring them more into line with the other datasets, removing genes expressed at very low levels where measurement error is higher, as described in the Materials and Methods.*

*For point (2), we agree with the reviewer that alternative splice variants were not controlled for in our original manuscript or in any other of the published models that explain translation rates (TR). We agree that this lack of control in effect introduces error in the translation rate data which will reduce the effectiveness of any model in predicting TR. However, our key conclusion is that the general features as we have defined them control much more of TR than any prior study suggested, and this conclusion is not undermined by this potential source of error due to alternative splicing. We had acknowledged in detail at the beginning of the Discussion that error in the TR data may result in underestimation of the role of the general features. Moreover, our discussion of possible sources of error in models is more comprehensive than any we recall reading in other similar studies.*

*Indeed, one of our conclusions could be impacted by differing degrees of error due to variations in transcript structure. We note in the Discussion that the general features are less predictive of TR in metazoans than in the two yeasts. Here one could imagine that isoform complexity may result in more error in metazoans, which have more mRNA variants. But we suggest that it is more likely that the large differences in the numbers of miRNA and RNPs are the cause, consistent with general thinking in the field. In addition, our analysis of one general feature, codon usage, agrees with the work of the population genetics community, who have established that codon biases and effective populations sizes are larger in yeasts than in metazoans, results that do not rely on ribosome profiling or mRNA expression data. The greater role we see for codon usage in controlling TR in yeasts than in metazoans matches the results from the population geneticists, giving us confidence that the differences we see are not solely due to differing error in the TR data.*

*It is technically impossible to accurately determine the translation rates of each individual mRNA splice variant. There is no deterministic way to know which variant each short read maps to, as the reviewer acknowledges. Moreover, there is a lack of tissue/condition specific mRNA splice variant annotations. For this reason, we cannot accurately infer the translation rates of mRNA splice variants in a probabilistic/statistical way. Instead, we have adopted an alternative, clear-cut approach in this revised submission. We have taken the datasets from Arabidopsis and mouse and limited the genes in each to a set that have only a single annotated mRNA isoform. We find that for these single isoform genes our model’s predictive power is similar to that for the complete (complex isoform) set of genes. For some datasets predictive power is a little higher for the single isoform genes, for others a little worse. This rules out any substantial error in our conclusions due to isoform variation. We have included this analysis in the Results section associated with Figure 13 (see Figures S10 and S11). We thank the reviewer for spurring us to get around to performing this control as we know others in the field have raised this possibility as well. This is a useful improvement to our manuscript.*

*We finally note that the heterogeneity of splice isoforms can be over emphasized: Most genes have a single predominant splice variant in a given cell line (85% of the abundance of human cellular transcripts are comprised of the predominant variant) (Gonzalez-Porta et al 2013, Genome Biology); and most splice variants that are actively translated (i.e. those that are not aberrantly spliced—intron retained—variants that are subject to NMD decay) encode a common CDS sequence (Reyes and Huber, 2018, NAR).*

2) The genome annotations in metazoans are much more complicated than those in simple eukaryotes like S. cerevisiae and S. pombe. The authors don't describe in the main text which annotations were used or how 5' UTR sequences were obtained. Indeed, many genes have alternative 5' UTRs and it would be vital to use the 5' UTR isoforms expressed in each experiment for correlative analysis with the corresponding ribosome occupancy. This also underscores the importance of consistent data processing.

*RESPONSE*

*We had included the exact sequences of the mRNAs used in our analysis in Additional File 2 as we believe that is the only unambiguous way to define the data used. We have now added to the Materials and Methods a statement about the sources of the mRNA sequences used in the analysis. These are either identical to those used in the original publications or only include minor technical changes in genome annotations, allowing us to use a single annotation version for each species. Since the original publications are highly regarded, we are confident in using the same (or highly similar) annotations in our work. Please also see above for our response to the suggestion of consistent data processing and how we have now shown that isoform variation is not a major source of error.*

3) Datasets from different species have differing amounts of polyA bias. The Weinberg S. cerevisiae dataset has essentially no polyA bias by using Ribozero to estimate mRNA levels. This led to very different ribosome occupancy estimates. Indeed, it decreases the translation efficiency (ribosome occupancy) variance among genes in S. cerevisiae. What about the other datasets? Do they have more apparent variance among translation efficiency? This might help explain why less of the variance is captured by the regression models for the other species and datasets, compared to the Weinberg data.

*RESPONSE*

*Like the Weinberg S. cerevisiae dataset, the other datasets we have used do not have a significant poly A selection bias. S. cerevisiae is a special case because, out of the species/tissues we have examined, it suffers much greater RNA degradation during RNA extraction. Figures 1a and S1 show that the variance in TE values for the “good” S. cerevisiae data is comparable to that of the other datasets, implying that they are all of similar quality. We had included these figures to help readers get a feel for the data.*

*Of the two S. pombe datasets, one (#1) was poly A selected while the other (#2) was ribo off processed. Of the Arabidopsis datasets, the shoot 2 dataset was poly A selected whereas the shoot 1 and root 1 datasets were ribo off processed (NB the sample labelled “leaf” in the original submission should have been labelled “shoot 2”). There are other differences in the protocols used by the groups who generated these data as well. Despite these differences, the distribution of TE values are very similar between the poly A selected and ribo off datasets for a given species (Figure S1). Our model predictions of TR are a little different for poly A selected and ribo off datasets, but these small differences do not exhibit a clear trend that suggests an effect of polyA bias: S. pombe #1 R2=0.53 vs # 2 R2=0.65; Arabidopsis shoot 2 R2=0.45 vs root 1 and shoot 1 R2=0.37 & 0.40.*

*We have discussed the poly A selection bias with Stephen Eichhorn, who produced much of the ribosome profiling data from the Bartel lab. for many species. Both he and David Weinberg assured us that the RNA degradation / poly A selection issue is only a major problem in S. cerevisiae, a view that our analysis supports.*

4) Ribosome profiling does not provide translation efficiency estimates. It provides ribosome occupancy estimates only. The dataset from S. cerevisiae tried to account for this by taking into consideration codon usage. It has Initiation Rates estimates, while the other datasets have ribosome occupancy only. Even so, one can't exactly equate the two things. The authors should note this caveat somewhere in the introduction or results.

*RESPONSE*

*We had noted this. In the first paragraph of the results we had stated that “Translation rates were determined from ribosome profiling data as prior work has shown that the density of ribosomes per mRNA (i.e. the translational efficiency) is a useful estimate of the rate [13, 23].” We have now added the word molecule to further clarify the point: “that the density of ribosomes per mRNA molecule”. We hope that this is sufficiently clear.*

*We also note that the S. cerevisae adjusted IE values are very similar to the unadjusted TE values (R2=0.9), so the adjustment is minor anyway. In addition, given that codon bias is much reduced in metazoans compared to S. cerevisae, it is expected that IE and TE values will be even more highly correlated in plants and animals.*

5) As above, the yeast dataset has codon usage baked into the "initiation rates" (Weinberg et al), while the other species datasets do not. Does this account for the fact that the models recover more of the variance in yeast data? Since the impact of codon usage is already included into the initiation rate estimates, is it fair to use codon usage as a predictor for these initiation rates in S. cerevisiae?

*RESPONSE*

*We believe it is fair. In fact, the calculation of IE values involves the reduction of codon frequency effects, so that the final IE values can better capture ribosome density per codon. Hence, the impact of codon usage is not included but rather excluded from the IE values. We also note that codon usage makes a slightly larger relative contribution to the unadjusted TE values for the other yeast, S. pombe, than it does to the IE values of S. cerevisiae, and a smaller contribution to the metazoans. These results are consistent with the population genetics community's data on effective population sizes and codon bias.*

*6) The authors use "translation rates (e.g. page 20, Line 11) when they mean translation efficiency.* "Translation rates" are the number of proteins translated per unit time. Translation rates (estimated by ribosome occupancy) correlate quite well with protein abundance (in contrast to author's claim on pg 20 line 12). I realized later on that they defined "translation rate" in their earlier NAR publication this way, but didn't see it defined in this manuscript.

*RESPONSE*

*Unfortunately, the literature is inconsistent in how to define “translation rate”. It is sometimes used as the reviewer suggests to mean the total number of protein molecules synthesized per gene per unit time, though “protein synthesis rate” is also used in this context. Translation rate is more widely understood—as we use it—to be the number of protein molecules synthesized per mRNA molecule per unit time. We had stated at the beginning of the Results that ribosome profiling TE values are a fair approximation of translation rates, defining these as “the density of ribosomes per mRNA”. We have now clarified this further by adding the word “molecule”. i.e. “the density of ribosomes per mRNA molecule”. Our first sentences of the Introduction also makes it clear that we are considering the translation rate per mRNA molecule: “It is a major challenge to determine from nucleotide sequence data the rates at which eukaryotic mRNAs are translated into protein. There are two classes of cis-acting elements in mRNAs that determine these rates: general sequence features and gene/condition specific elements [1-11]. The general features are secondary structure in the 5’ portion of the mRNA; upstream open reading frames (uORFs), which lie 5’ of the protein coding sequence (CDS); specific nucleotides immediately flanking the initiating AUG codon (iAUG) at the 5’ of the CDS; CDS length; and codon usage.” It would make no sense for these mRNA sequence features to define a substantial proportion of the total number of protein molecules synthesized per gene as that is largely determined by the abundances of mRNAs, and thus largely determined by transcriptional control signals. From the introduction alone, we believe that readers should understand that we mean the translation rate per mRNA molecule.*

*In addition, we worry that it would be confusing to general readers to alternate between TR and TE in the text as if these are two different things. TE is a technical term only used by a subset of researchers, typically just to refer to that metric from ribosome profiling data. A wider audience understands what a translation rate is. No method is an exact measure of TR. IE, TE, and MS measured protein synthesis rates per mRNA molecule are all approximations of true TR.*

7) The AUG Proximal Element regions (APEs) vary too much between species to allow meaningful across-species comparisons. Typically, this region is thought to comprise the "Kozak" sequence (-5 to +3), but the authors have allowed it to occupy much more sequence in S. cerevisiae and nearly the entire UTR region in A. thaliana. How do the results change if you consider only the Kozak region, or same sized regions as APEs?

*RESPONSE*

*Our Figures 7 and S4 had answered this question. Please look at the right hand most purple data point for -5, 5’UTR only (sequences +1 to +3 are identical in all genes and thus contribute no explanatory power). This question can be answered by comparing the predictive power for this data point among the different species. For example, in S. cerevisae a PWM for the Kozak region determines ~8% of TR whereas a PWM of the APE determines 24%. These values, though, underestimate the total contribution of each region as they do not include the additional impact of di- and tri-nucleotide frequencies. Our BIC selected, multivariate APE model explains 33% of TR, a considerable improvement over the 8% explained by the Kozak PWM.*

*Remember that Marilyn Kozak’s original consensus was designed to distinguish the initiating AUG from other AUGs in mammals, and was not initially intended to determine differences in the rates of translation at initiating AUGs across mRNAs. Dr. Kozak did not have translation rate data or the depth of sequences we now have. We did not intentionally designed our APEs “to occupy much more sequence”. Instead, we used an objective approach to decide the APE boundaries that optimize the prediction of translation rates. Our results show that the APE is only approximated by the Kozak consensus in mammals and S. pombe, and even in these species the Kozak element fails to capture additional sequences +4 to +7/+13. In Arabidopsis and S. cerevisiae (and Drosophila) the APE is clearly much larger than the Kozak consensus.*

*We agree with the reviewer that the different lengths create a difficulty in comparing the contributions of APEs, but we find no objective justification for limiting the analysis to the short, historic Kozak region given our results. There is no perfect way to compare the contributions of APEs, but at least we have used criteria that are objective.*

*We have now added a specific mention of the Kozak consensus in the results as both reviewers have suggested it: “Arabidopsis has, like S. cerevisiae, an extended APE, spanning nucleotides -65 to +33, whereas S. pombe and the two mammals have shorter APEs that span -6 to +13 or less and in which the Kozak consensus [34] plays a major role.”*

Discussion section issues:

8) The higher G/C content in mammalian 5' UTRs might be explainable simply as a difference in growth temperature, as the other species must accommodate shifts in growth temperature that alter the stability of 5' UTR structures (especially at low temperature), while mammals maintain a constant, relatively elevated temperature. This could be discussed, but may have been covered by previous work (e.g. by Michael Lynch).

*RESPONSE*

*We agree that it is interesting to consider why mammalian genomes have higher GC contents, but we find it a little off topic to include a discussion of this point in our Discussion, because this point is not directly related to translation rates. Given the many pertinent points we have included in the Discussion, we hope that the reviewer will agree with us.*

9) It is true that RNA abundance correlates fairly well overall with protein abundance. However - codon usage influences mRNA abundance by affecting RNA decay rates (Coller, Green, and others). It is thus wrong to claim that translation rates correlate poorly with protein abundance and that transcriptional control is the primary influence. Furthermore, recent work has linked RNA decay with upregulated transcription in mice and zebrafish (Nonsense Induced Transcription Compensation, Wilkinson et al. N&V, 2019), echoing older work from yeast (Choder lab). Thus transcription rates are influenced by translation rates. The connections between these processes complicate comparisons of the influence of transcription and translation on protein production. The authors should present a more balanced view on this topic in the discussion, describing such caveats.

*RESPONSE*

*We wish to assure the reviewer and the Editor that we do not have a bias against translation. In fact, we have and are continuing to devote considerable efforts to understanding control of this fascinating process. It is just that our data suggest answers that are different from those most commonly considered. Based on our understanding, the reviewer would like us to include a more detailed discussion of points already made in other papers that we reference. It is standard practice, however, to cite prior conclusions without replicating detailed justifications given in earlier work. We cannot re-write a review on this topic here. Instead, we did reference a Perspective we published in Science supporting our claims, as well as other work.*

*When we cite that transcriptional control is dominant over translational control, this is based on objective, quantitative ANOVA studies, including our own, that also take into account RNA turnover as well as the evidence that translation directly accounts for only 8% of protein abundances. RNA decay also only explains ~8% of protein abundance, so even if codon usage explained all of RNA turnover, then the most that translation could explain is ~8+8=16% of protein abundance. In fact, even in S. cerevisiae (where codon bias is strongest), codon usage explains only ½ of RNA turnover (cheng et al 2017. RNA 23, 1648). So perhaps 12% of protein expression could be explained by translation in this yeast. (There is a sophistication beyond these metrics, however. Our previous NAR paper contains the details.) As to transcription compensation, there is almost no correlation between RNA turnover and transcription rates. This means that any such feedback can play only a very limited role determining the differences in mRNA levels between genes at steady state. It is possible that such feedback may play bigger roles in some specific genes, but our work focuses on genome-wide results at steady state, and we are just quoting the results of the most careful, quantitative genome-wide studies.*

10) Also, statements about the lack of importance in translational control made in the discussion are based entirely on correlative analyses of steady-state measurements in single-cell organisms and tissue culture cells. Translational control is important for organismal development, meiosis, local protein production (e.g. at the ends of neuronal axons), immune responses, general stress responses, etc. The authors should note their results are based on steady-state cell growth conditions, and thus miss many aspects of translational control somewhere in their discussion, which currently comes off as biased against any important role for translational control in biological organisms.

*RESPONSE*

*We fully agree that our statements are limited to the situations we have studied. However, we did not only look at single cell organisms and tissue culture cells as the reviewer suggested. We also looked at whole tissues: plant four day old seedling root and shoot (i.e. an entire organism); and mouse whole liver and whole kidney. Please see Figure 13, which shows these results. We have made the tissues used clearer in revised text. “Results were calculated for the five example datasets (S. cerevisae, S. pombe 2, Arabidopsis shoot 2, M. musculus NIH3T3 cell line, and H. sapiens HeLa cell line), for a second S. pombe dataset (S. pombe 1), and for data from additional tissues or biological replicas for Arabidopsis (root 1 and shoot 2) and M. musculus (whole liver and whole kidney).”*

*We of course agree that there may be differences in translational control in some cells. But in this manuscript we have looked at a wide sampling of species, tissues and cancerous states and find similar results in all cases. That implies that the principles we have uncovered are quite general, though of course not necessarily universal, see below.*

*We have added a statement indicating that current studies are limited to just a few conditions. “In contrast, the selective pressure on protein abundance dominantly affects transcription, at least for the few conditions for which this has been most carefully measured by Analysis of Variance (ANOVA) [10, 61].”*

*For the record, we have already used our pipeline to uncover dramatic differences in general feature control in the oocyte to embryo transition in Drosophila, providing support to the reviewers idea. Since this analysis involves another 10 datasets (developmental stages and mutant backgrounds) and is intended to be link to additional ones from Xenopus and Zebrafish, we hope that the reviewer will understand why we cannot include this work in this current publication.*

12) The evolutionary arguments in the discussion are also highly biased against translation and don't consider alternatives. For example, "...the length of a protein is unlikely to be selected only to affect translation. Protein length is presumably set largely by protein function" (pg 20 line 13). The opposite point could be argued. Housekeeping proteins are small, often splitting single functions into multi-domain complexes. One benefit of keeping such proteins small is that they are more efficiently translated. Similarly, "Translation rates are determined by multiple selective pressures, In contrast, the selective pressure on protein abundance dominantly affects transcription"(pg 21 line 1). One could argue that mutations that decrease translation rates have fitness effects and many features of 5' UTRs are highly conserved.

*RESPONSE*

*In the revised version, we have noted that it is indeed possible that the most highly abundant proteins (e.g. ribosomes and histones) are selected to be small to allow efficient translation. (We left this sentence out in our previous submission because some found it a confusing aside). We have now put that comment back in:*

*…… “Second, while CDS length is an important determinant of translation rates (Figure 13), the length of a protein is unlikely to be selected only to affect translation. Protein length is presumably set largely by protein function. There is a caveat to this, though: it is plausible that the most highly abundant proteins that are members of multi-subunit complexes, e.g. histones and ribosome, are selected to be short to allow efficient translation.”*

*The above notwithstanding, we still think that the selective force of translation is unlikely the major determinant for the length of the vast majority of proteins, which are present at much lower levels and thus not individually able to affect cell efficiency. (Remember that the hundred most abundant proteins account for 50% of total protein mass in a cell, which means that the selective force for efficiency on the other 5,000 - 10,000 proteins in a cell is small. Further—in a sophistication beyond the scope of this current manuscript—the component of the variance in TR that is independent of mRNA abundance is strongly dependent of CDS length in S. cerevisiae, more so than the mRNA abundance dependent component, see our NAR paper).*

*Surely it would be biased to say that all proteins are the length that they are solely to control the rate of translation? That would imply that protein length has nothing to do with the function of a protein, a quite radical idea.*

*We do not find our statement that “translation rates are determined by multiple selective pressures” as “highly biased” or not “considering alternatives”. We had clearly acknowledged that there are multiple explanations; we did not only discuss one possibility.*

*When we say “In contrast, the selective pressure on protein abundance dominantly affects transcription" we are referring to ANOVA analysis, not to a population genetic studies of selection. We have added a clarification to this sentence in the revised manuscript: “at least for the few conditions for which this has been most carefully measured by Analysis of Variance (ANOVA).”*

12) Underscoring the counterarguments against the evolutionary statements in the discussion, She and Jarosz recently mapped hundreds of causal nucleotides responsible for natural phenotypic variation in yeast, and found that causal variants in 5' UTRs, Synonymous changes, and 3' UTRs were more numerous than variants in intergenic regions (Cell 2018). Mutations that introduce uAUGs are linked to numerous human genetic disorders. Thus there is ample evidence for translation efficiency variation affecting phenotype and being subjected to negative selection.

*RESPONSE*

*We thank the reviewer for mentioning these other important studies; however, we do not think they argue against the points we make. First, She and Jarosz have not conducted an unbiased genome-wide study but have instead found several hundred loci that are implicated by one of five drugs or other selective regimes. It is difficult to know how their finding relates to genome-wide control. Second, the reviewers’ description of their work is inaccurate. She and Jarosz found only one 5’UTR mutation that compellingly explains phonotype (i.e. that might affect translation), whereas they found more promoter mutations, which the reviewer did not mention. Third, most mutations that She and Jarosz found are actually missense, emphasizing that they are mostly not looking at cis-control but at protein function. Fourth, the phenotypic assay used by She and Jarosz is very complex and open to a variety of interpretations. For example, the statistical approaches used in this pioneering study are new and have yet to the widely accepted. By contrast, we and others have used more direct measurements of control and have used ANOVA and other well established statistical methods.*

13) The authors results suggest that single nucleotide variations in 5' UTRs could drastically shift translation efficiency (pg 17, line 3) by altering RNA structure. This also supports the above arguments that changes in 5' UTR sequences likely alter phenotypes and are likely subject to natural selection.

*RESPONSE*

*We make a quantitative argument about the degree that each step impacts protein abundance. All steps have some effects, therefore all steps can affect fitness. We do not in any way suggest that translation plays no role in controlling protein levels.*

*Many people currently assume that translation has to be about changing protein abundance and nothing else. We argue (based on clear data) that this is only a small part of its function and that it plays additional roles. We are not arguing that some roles are unimportant. Just that translation plays multiple roles.*

14) Pg 21, line 16 "mutations" should be "sequences", as these were not changes from a wild type, but rather random sequences cloned in the 5' region.

*RESPONSE*

*We thank the reviewer for this correction. We have made the change to “sequences or mutations” because the study from the Segal lab. randomly mutated a single wild type sequence. We modified the wording in the remainder of the section to be consistent as well.*

15) Pg 22, line 11 "...measured endogenous translation rates". The authors should note that they aren't using translation rates, they are using yeast initiation rates estimated from ribosome profiling after accounting for codon effects.

*RESPONSE*

*We have addressed a related comment 6) by the reviewer earlier. We have used the term “translation rates” throughout, having indicated in the first paragraph of the results that actual measurements are an approximation.*

*We should point out that we are careful to distinguish measured rates (however measured) from true rates. We think this distinction is the more important one to make. It is also sadly one that many other papers failed to be clear about.*

16) The manuscript should be reorganized to reduce the number of main figures (17), and reduce the length.

*RESPONSE*

*We would like to keep the current length for the following reasons.*

*First, many of the two reviewers’ questions, for example, were addressed by figures included in the original submission, such as Figures 1, S1, S4, 7, and 13.*

*Second, we realize that we have combined the results from what could have been two or three papers into one. We did this because we find the work fits together and is best presented as we have it. We have made every effort to communicate clearly, balancing the need to provide sufficient detail to rigorously establish our claims while making the work as generally accessible as possible. Genome Biology is valued as a journal in part because it recognizes that one size does not fit all manuscripts. We present a challenging, sophisticated analysis of five diverse eukaryotes. The scale of our analysis far exceeds that in any prior study. Hence, we would not want to weaken it by reducing its strength or clarity.*

*If the reviewer has specific suggestions on sections or sentences that are unclear or redundant, we will gladly address them. While one could move some figures to supplements, in an online journal there is no reason to if they are important. We had already tried to put some limit on the number of main text figures, for example several of the supplementary figures are also critical to understanding, such as S2 and S10.*

Reviewer #2: Review of Biggen et al. "Quantitative Principles of cis-translational control by general mRNA sequence features in eukaryotes"

mRNAs encode many cis-regulatory elements that have the potential to control the rate of protein synthesis. Such elements include secondary structures, uAUGs & uORFs, start codon context, CDS length, and codon usage. While the role of individual elements has been demonstrated on particular mRNAs, computational models that integrate each element to account for differences in translation efficiency are lacking or have poor predictive power. Here Li et al report an integrated model that accounts for about 40-80% of the translation rate (TR) variance in five species: S. cerevisiae, S. pombe, A. thaliana, M. musculus, and H. sapiens. They demonstrate that the stability of the most stable secondary structure in a 5' region of a gene has predictive power for TR across all species except S. pombe

*RESPONSE.*

*Actually the most stable structure has the largest predictive power in all five species.*

The location of the most stable secondary structure varies among species, either near the 5' cap (mammals) or the start codon (the others). They further show that across all five organisms, changes in tri-nucleotide frequencies correlate with TR. By integrating models that account for the 5' region features, codon frequency, and CDS length they yielded a model that explains 37-81% of the variance in TR. Lastly, they demonstrate that 5' region features, codon frequency, and CDS length are collinear suggesting that these features are coordinated to control ribosome density on mRNAs.

Despite its potentially interesting findings and its clarity, I cannot recommend publication of this manuscript in Genome Biology at this point. One hesitation is that they have previously published a model (Li et al. 2017. NAR.) that accounted for 80% of the TR variance in S. cerevisiae. It is unclear how the model/approach they present here is different from their previous work since the reported predictive power is identical to the what they have previously reported for S. cerevisiae. My interpretation is that it is quite similar or even identical.

*RESPONSE*

*Our current work differs from our NAR paper in two main aspects. First, our new model is a tremendous improvement on the preliminary one published in NAR. Second, our current manuscript contains considerable additional analyses that are unique to it and go beyond the model. All of the major conclusions in this current manuscript are unique to it and not to our earlier NAR publication, which was focused only on S cerevisiae and on a decomposition of translation rates to provide valid metrics for the control of protein abundances.*

*The largest difference between models is for RNA secondary structure. In our NAR paper, we used just one feature: the mean energy of a series of 35 nucleotide windows for the 5’UTR. In our current paper, we tested 421 features that include 21 window lengths and many ideas that probe what aspects of RNA structure control TR. Our original NAR model explained 19% of the variance in TR in S cerevisiae, our new one 33%. Our old model only explained 3% of the variance when we (in this current paper) tested it on mouse, but our new model explains 17%, a >5 fold improvement (see Figure 3). But the most important improvement in our new model is that it shows mechanistically the aspects of RNA structure that control translation rates, whereas our earlier model had nothing to say on this subject. RNA structures affect other processes such as RNA degradation and splicing. It is quite plausible that the greatly improved metrics for RNA structure in our new model will be helpful in predicting and understanding control of these processes too.*

*Further, our model for uAUGs has been changed in this current publication to make it general to all eukaryotes. Also, our NAR model did not include the 5’ofAPE region, whereas our new model does. This is a critical improvement for predicting TR in mammals as the 5’ofAPE has over twice the predictive power of the APE in mammals. Only the APE, CDS length and codon usage feature sets for S cerevisiae replicate those in our earlier NAR publication.*

*One should not judge a model only by the percent variance explained. Instead, the true purpose of a model is to provide a genuine understanding of process, a point that is sadly missed in the “black box” models that have proliferated in the literature. In this regard, our new model and the associated analyses, such as the extensive collinearity we show uniquely in this paper, are a great advance on the preliminary effort shown in a few figures at the end of our NAR paper.*

*Finally, in addition to our improvement on the model, another major contribution of our work is demonstrating that many of the TR control principles in S cerevisiae (discovered in this current manuscript) are also true in another yeast, a plant and two mammals. We believe that many interested in the system wide control of protein abundances or in decoding eukaryotic genome sequences will be fascinated by such a cross-species study.*

For instance, the S.cerevisiae data included in Figure 7 appears identical to data they have previously published (Li et al. 2017. NAR, Figure 8A,C). If so, this must be qualified and cited.

*RESPONSE*

*We apologize for this confusion. In fact, as we had indicated in our text, the few instances where we showed replicas of our NAR results for S cerevisiae to allow comparison to other species. In the specific example given by the reviewer, our text read and still reads…*

*“One feature we used previously to explain translation rates in S. cerevisiae exploited sequence differences between highly and poorly translated mRNAs without regard to the biochemical mechanism(s) of control [10]. This approach proved powerful, showing that nucleotides flanking the iAUG from -35 to +28—a region termed the AUG proximal element (APE)—explain a third of the variance in rates [10]. To extend this strategy to the other four species, we first compared Position Weight Matrices (PWMs) for sequences -80 to +35 for the most highly translated (high TR) and most poorly translated (low TR) 10% of genes (Figure 7a; Additional file 7).” ……………“Following the strategy used earlier for S. cerevisiae (see Materials and Methods), we assigned a score to each gene based on PWMs of varying lengths in high TR genes and defined APE boundaries by maximizing the R2 between the PWM scores and translation rates (Figure 7b; Additional file 1: Figure S4).” note that ref 10 is our NAR paper.*

*To our minds the above text is clear in indicating that our APE model was developed for S cerevisiae in an earlier publication and is in this current one being extended to four other species. However, we note that the APE model is only a component of our current, more extensive model for TR.*

Further, much of Figures 2-12 could be condensed and summarized as a few key figures in the main text, with much of it instead included in the supplement.

*RESPONSE*

*Similar to what we replied to Reviewer 1’s comment 16), we prefer to keep the current figure layout instead of losing critical information by condensing figures. Figures 2-12 present rigorous analyses and novel results that address fundamental questions. For example, Figure 11 shows a new approach to comparing mRNA sequences between species. What it reveals for the first time is that there is a remarkable conservation across distant species in the sequences that control translation. Standard approaches, such as looking for homology between aligned sequences or for matches to PWMs, have failed for the last two decades to see what is in fact a stunning degree of conservation. Hiding this fundamental discovery in a supplementary figure would be strange. As a second example, Figures 4, 5, and 6 collectively show that the aspects of RNA structure that control translation are remarkably similar across five very diverse eukaryotes. Prior to our study, it was only understood that RNA structures of some undefined kind affected translation. Now in this paper we have determined what the structures look like and that they are relatively similar across eukaryotes. We find the results in Figures 2–6 alone make a major contribution.*

I think the authors make interesting points that are obfuscated by the amount of analyses that surround them (particularly Figures 3 & 9). Summary diagrams that outline species specific findings would also be helpful.

*RESPONSE*

*We believe that our surrounding analyses are necessary because they show our points are not only interesting but also credible. Our more compelling points are those that are general to all or to several species, so we are afraid that figures that summarize species specific findings would highlight less compelling, less interesting results. Our current figures do give results for each species and in some cases multiple tissues/cancerous states, allowing readers to judge what variation is unique to a given species. The unusually broad scope of our paper means that some of the figures are large, but we have tried our best to make them easy to understand.*

It is interesting that a majority of the variance in TR in the examined single-celled organisms can be explained by the modeled general features, while only a minority is explained in multicellular organisms. Given their discussion points on the role of effective population size, the manuscript would be improved if the authors demonstrated that this observation held true for another non-fungal single-celled eukaryote. As is, the single-celled organisms they have profiled represent a different kingdom of life than the multi-cellular organisms they selected, which may limit the strength of their conclusions. One intriguing candidate would be Chlamydomonas reinhardtii, for which ribosome profiling data is available (PMID: 26286745) and would offer a useful comparison to Arabidopsis.

*RESPONSE*

*We thank the reviewer for this suggestion. Considering that no prior paper has looked at control in such a broad array of species and conditions as we have, we think the reviewer would agree that our work makes several profound discoveries. With all due respect to the reviewer, we think that adding one additional species, particularly a non-model such as Chlamydomonas, to our current work will not measurably increase its significance. Working on a less well characterized organism comes with plenty of downsides, such as less accurate genome annotations, even less choice of datasets etc., making us reluctant to go down that road. Moreover, since our figures are already large, squeezing in one more panel into each will further increase the complexity of figures.*

Further comments that should be addressed

1. The authors should describe how the example datasets were chosen, and whether the experiments were conducted using similar protocols (e.g. identical translation inhibitors, etc.). These data are the foundation of the manuscript and it is important to disclose potential experimental biases among the datasets.

*RESPONSE*

*As is standard practice in analysis papers, we did disclose the papers that produced the data, which of course describe in detail their protocols. We had included a section, Data and code, at the beginning of the Materials and Methods section detailing this. Hence, any interested readers can look up any issue that might interest or concern them. In the Data and code section, we also had described any averaging of results or filtering of the published data that we performed. We of course had also provided the mRNA sequences, TR and other data in Additional file 2 as well as our code and intermediate analysis files, so that our analysis results are totally reproducible.*

*The details of how the other researchers generated their data are as expected: they employed many of the protocol variants used in the field. Some used cycloheximide, others not. (Despite some differences in the small percent of reads that map to the very 5' end of mRNAs, inhibitors do not greatly affect overall TE values (e.g. Kronja et al. 2014 Cell Reports). Some prepared mRNA using poly A selection, others used ribo off. Different degrees of filtering were imposed on the data. One data set from each of three species was produced by the same graduate student (Stephen Eichhorn in David Bartel’s group), the other datasets were by others from several laboratories. The great strength of our analysis is that our main conclusions are robust across species and datasets and thus these protocol differences do not matter. We have now added the following general statement to the Data and Code section. “These data were produced by a variety of protocol variants in several laboratories. Our main conclusions hold across multiple datasets from different species, tissues, and protocol variants, suggesting that they are not influenced by the specifics of particular experimental methods.”*

*There was little to the choosing of the datasets; the selection available in some species is small. We took data from papers that had used the data to make cogent discoveries. We ensured that there was strong enrichment of footprints in the first positions of codons, and we only used datasets which have a positive correlation between TR and RNA abundance. The Weinberg S. cerevisiae data chose itself by being, arguably, the best controlled and validated in the field as well as the one we had studied in our NAR paper. Weinberg et al. showed that the other S cerevisiae datasets all had a poly A selection bias, so we avoided them. For S. pombe, we did not use an earlier dataset from one group since they advised us that their new ribo off selected one was a little better. We have added the following sentence to the Data and Code section: “Datasets were chosen in which ribosome footprints showed the expected strong enrichment for the first nucleotide in each codon and for which there was a positive correlation between translation rates and mRNA abundances.”*

2. In Figure 2 it is unclear why the authors chose to group S. pombe with S. cerevisiae and Arabidopsis given that High TR genes appear to have more stable secondary structures from +1 to +30, which contrasts with the other species. They should comment on this, especially since on page 6 lines 12-14 they state "Given this and a lack of evidence that RNA structure affects the elongating ribosome, we have limited our models to the 5'UTR and the 5 most part of CDS, where folding energy values and translation rates correlate positively." For S. pombe, it appears that more stable secondary structures just downstream of the start codon are correlated with higher translation efficiencies.

*RESPONSE*

*We did explain why we group S. pombe with the other two species in the text, and we repeat it here. “For S. cerevisiae, S. pombe, and Arabidopsis the differences between the two cohorts are largest from -35 to +35, -35 to +1, and -120 to +35 respectively. For mouse and human, the differences are largest towards the 5’ cap.” We also know, and reveal later in the paper, that S pombe, Arabidopsis and S. cerevisiae have the same A rich sequences just 5’ of the iAUG, supporting our choice of grouping.*

*For the second part of the reviewer’s comment above, there is some confusion. In S. pombe, more stable RNA structures (more negative delta G values) are correlated with lower translation rates (low TR). We make the comment about not using RNA fold energy differences downstream of +30 precisely because we want to exclude those sequences in S. pombe. While in S pombe an intuitive hypothesis might be that RNA secondary structure in the bulk of the CDS regulates translation, there is no biochemical evidence supporting that idea. Therefore, to be conservative we removed these sequences from our RNA folding energy model. (If we include those sequences, not surprisingly we make a more predictive, but not necessarily a more biochemically accurate, model).*

3. Figure 3 should be distilled, with most of the data included in the supplement. While the authors refer back to this figure at different points in the results, it is initially overwhelming and quite difficult to interpret. I would suggest a single R2 feature vs TR plot for each species that displays "whole", "RNA fold", and a subset of the previously used models as a comparison.

*RESPONSE*

*We thank the reviewer for this suggestion, but we prefer to keep Figure 3 as it is for the following reasons. First, as the reviewer pointed out, the details in this Figure are important for our main text. Figure 3 is a powerful new way to probe the functions of RNA structures. We have found it very helpful in other contexts as well: for example, visualizing how control by RNA structure changes dramatically during the fly oocyte to embryo transition. Since in advance one does not know what will be changed, it is important to look at all of the features. Second, we did something similar to the reviewer’s suggestion in Figure 4 by pulling out a small subset of the results from Figure 3, re-scaling the data to allow a cross-species comparison of one aspect of the analysis.*

*Please note how Figure 3 makes dramatic the similarity between species. Mouse and Human look like exact replicas for 421 features. S. pombe and Arabidopsis are also clearly related to the mammals, and S. cerevisiae more faintly. We find it impressive and compelling that so many criteria agree.*

4. Figure 7a. The y-axis label should be "probability" rather than "bits".

*RESPONSE*

*Thank you for noticing this mistake. We have changed bits to frequency.*

In these plots it is difficult to visualize the differences between the high and low groups among species. It may help to generate PWMs of high TR groups and use the low TR groups as the background, and vice versa. This would highlight enriched features in each group relative to the other.

*RESPONSE*

*We thank the reviewer for this suggestion, but cannot find a good way to implement the reviewer’s suggestion graphically. We believe that the current Figure 7a does demonstrate the points we wish to make. The much thicker band of blue and yellow (G and C) in low TR cohorts than in high TR cohorts is obvious in the yeasts and Arabidopsis. For the mammals, the point is that there really is no compelling difference at this mono nucleotide level between high and low TR cohorts. The figure does allow one to see that the mammals have a much higher GC content than the yeasts or Arabidopsis, though. That is all we wish to convey.*

Also, the authors don't seem to account for or acknowledge the potential role of the Kozak sequence within the APEs (Results, Sequence motifs in 5' UTRs). I suspect this will account for at least some of the variance in TR. For instance, in high TR genes from S. cerevisiae adenosine is enriched at the -3 position (relative to AUG), which matches the canonical Kozak sequence in this species. The same is true for S. pombe, but it appears less so for the mammalian species. While they allude to the Kozak sequence (I think) later on page 17 line 23, it would be helpful to discuss it during the section with the enriched sequence elements.

*RESPONSE*

*The role of the Kozak sequence can be seen in Figure 7b, the right hand most purple data point. This uses a PWM from -5 to -1. Indeed, in mammals and S pombe the Kozak sequence does contribute a high percent, but not all, of the explanatory power, which is consistent with the APE boundaries shown in Figure 7a. We have now added an explicit mention that the APEs in mammals and S pombe are strongly determined by the Kozak region:*

*“Arabidopsis has, like S. cerevisiae, an extended APE, spanning nucleotides -65 to +33, whereas S. pombe and the two mammals have shorter APEs that span -6 to +13 or less and in which the Kozak consensus [34] plays a major role.”*

**Second round of review**

**Reviewer 1**

The revised manuscript clarifies a number of issues that were present in the first submission. I am comfortable with the authors use and comparison of datasets from different species. Despite the caveat of alternative isoforms and short ribo-seq read lengths, the demonstration that similar predictor features were obtained using only single isoform genes is encouraging. Similarly, the authors explanations regarding my concerns with the discussion section address most of these issues adequately. The authors are incorrect in their response to my comment #12 regarding She and Jarosz Cell 2018, in which they claim that only one 5' UTR mutation was found that affected phenotype. She and Jarosz don't give a specific count, but from their Figure 4B, one can estimate that the 3-4% of all causal variants that are in the 5' UTR represent 11-12 mutations (of the 370 total causal variants). Causal synonymous variants are also more frequent than intergenic (presumably promoter) variants in that figure. The point of my original concerns was that the authors make a number of evolutionary arguments in the discussion section that I interpreted as suggesting that selection on protein abundance doesn't affect translation rates, when She and Jarosz provide clear evidence that synonymous codon sequences, 5' and 3' UTRs all affect fitness and could thus be subject to natural selection. I believe the authors did not initially mean this, that I had the wrong impression from their original submission. The revised discussion better highlights the significant novel results from the manuscript while reducing the appearance of biases against translational control.

The main remaining challenge with the manuscript is the number and format of figures. I agree with the second reviewer that these should be condensed. It is unfortunate that the authors have made no attempts to remedy this issue in the revision. Although Genome Biology is an online journal, many researchers read articles in pdf format and casual readers from related fields are apt to browse figures. Currently, the 17 included figures are unwieldy and the manuscript would receive more comprehension and attention from readers by reformatting. Figure 1 could be supplemental. Figures 2/3 could be summarized more succinctly in a single figure. Figures 4-6 are all single panel and could easily be merged. Figures 7 / 8 could be merged, etc. This would be in both the interests of the journal and the authors, as the manuscript would be more readable and likely garner more citations.
